# Duodenocaval fistula after bevacizumab therapy: case presentation and complete overview of the literature

**DOI:** 10.1093/jscr/rjad329

**Published:** 2023-06-10

**Authors:** Alessio Giordano, Francesco Moroni, Alessandra Gosmar, Federico Passagnoli, Juhye Jeong, Francesco Menici, Maddalena Baraghini, Vita M Mirasolo, Laura Campiglia, Stefano Michelagnoli, Stefano Cantafio

**Affiliations:** Surgery Department, Santo Stefano Hospital, ASL Toscana Centro, Prato, Italy; Surgery Department, Santo Stefano Hospital, ASL Toscana Centro, Prato, Italy; Surgery Department, Santo Stefano Hospital, ASL Toscana Centro, Prato, Italy; Surgery Department, Santo Stefano Hospital, ASL Toscana Centro, Prato, Italy; Surgery Department, Santo Stefano Hospital, ASL Toscana Centro, Prato, Italy; Surgery Department, Santo Stefano Hospital, ASL Toscana Centro, Prato, Italy; Surgery Department, Santo Stefano Hospital, ASL Toscana Centro, Prato, Italy; Surgery Department, Santo Stefano Hospital, ASL Toscana Centro, Prato, Italy; Intensive Care Unit, Santo Stefano Hospital, ASL Toscana Centro, Prato, Italy; Surgery Department, Vascolar Surgery Unit, S. Giovanni Di Dio Hospital, ASL Toscana Centro, Florence, Italy; Surgery Department, Santo Stefano Hospital, ASL Toscana Centro, Prato, Italy

## Abstract

Duodenocaval fistula (DCF) is a very rare condition and is associated with a 41.1% of mortality rate. Although ingested foreign bodies, peptic ulcer disease and radiotherapy are often the etiologies described, only three patients have been described who developed DCF after bevacizumab therapy. We report a case of a 58-year-old woman with a history of ovarian neoplasia and subsequent surgical treatments, adjuvant radiotherapy and chemotherapy with bevacizumab with the appearance of a spontaneous DCF after 6 months at the end of this therapy. The multidisciplinary approach between oncologist and vascular surgeon together with the support of the anesthesiology team allowed the DFC to be treated surgically through the suture of the inferior vena cava and the duodenal breach. The patient was discharged on the 14th postoperative day and we found no postoperative morbidities both immediately and after 30 and 60 days.

## INTRODUCTION

Duodenocaval fistula (DCF) is a rare life-threatening disease, with only limited literature available regarding its treatment. It is a very rare and at the same time very lethal condition in which a direct one is created communication between the inferior vena cava (IVC) and the duodenum. This entity is most commonly found in men in the fifth decade of life and is related to a mortality rate of up to 40% [1]. The typical presentation has been gastrointestinal hemorrhage or sepsis. The most common etiologies include trauma, migration of an IVC filter, ingested foreign bodies, peptic ulcer disease and retroperitoneal tumor resection combined with radiotherapy or chemotherapy. The high mortality was attributed to the difficulty of establish a diagnosis that can quickly guide an appropriate therapeutic intervention or to initial clinical manifestations, which in some cases can be very sudden and lead to the death of the patient.

We report the case of a 58-year-old woman with a history of ovarian neoplasia and subsequent surgical treatments, adjuvant radiotherapy and chemotherapy with bevacizumab with the appearance of a spontaneous DCF. This is the fourth case described in the literature.

## CASE REPORT

**Figure 1:**
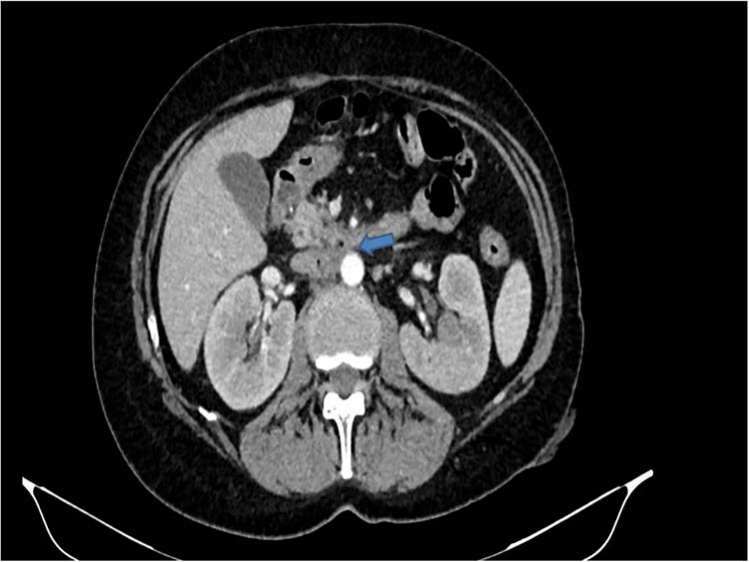
CT scan showed the presence of strong adherence between the IVC at the origin of the left renal vein and the third duodenal portion.

**Figure 2:**
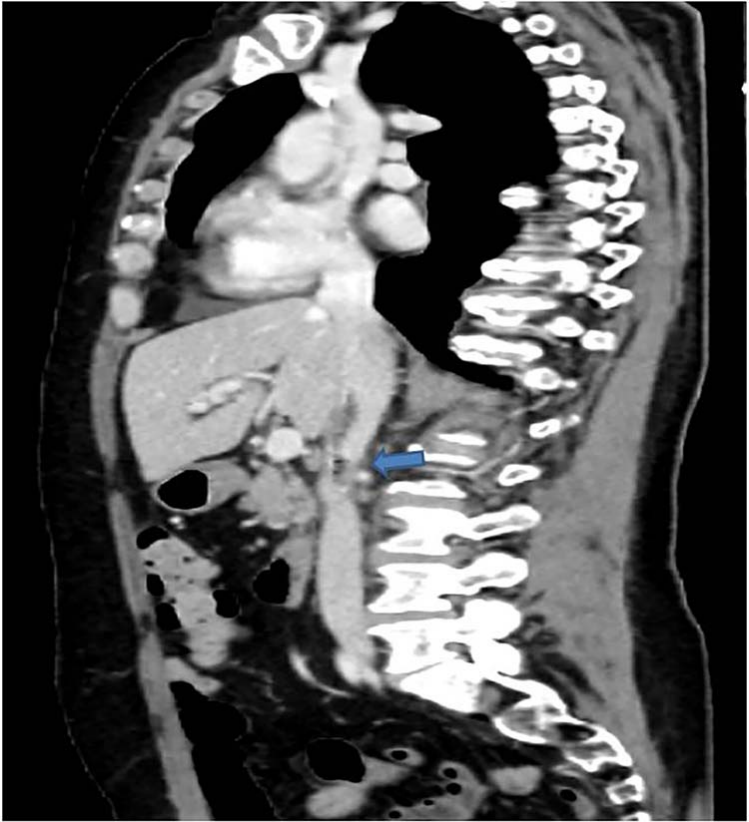
In coronal view the arrow points the air inside the vena cava and thrombotic-like hypodense material.

A 58-years-old woman arrived to our emergency department for fever (39.5°C) with chills and diffuse abdominal pain radiating to the back about 2 weeks. Her medical history included a hysteroannexectomy with sigmoid resection for an ovarian tumor that occurred 2 years earlier (Stage IIb) and adjuvant radiotherapy and traditional chemotherapy, subsequently followed by biological therapy with bevacizumab completed 6 months earlier. The patient was currently disease free and in follow-up. On admission, laboratory test showed a white cell count of 18 700 mL and microcytic anemia (hemoglobin level, 6.6 g/dL) with an increase in inflammation indices (CRP 26.2 mg/dl). Evidence of several digestive tract bacteria, including *Enterococcus faecium*, *Escherichia coli* and *Weissella confusa*, was disclosed by means of several hemocultures. A specific antibiotic therapy was started and blood transfusions were performed for the anemia found. Therefore, the patient underwent a chest and abdomen CT scan, which showed the presence of strong adherence between the IVC at the origin of the left renal vein and the third duodenal portion with air inside the vena cava and thrombotic-like hypodense material as for a DCF (Figs 1 and 2).

**Figure 3:**
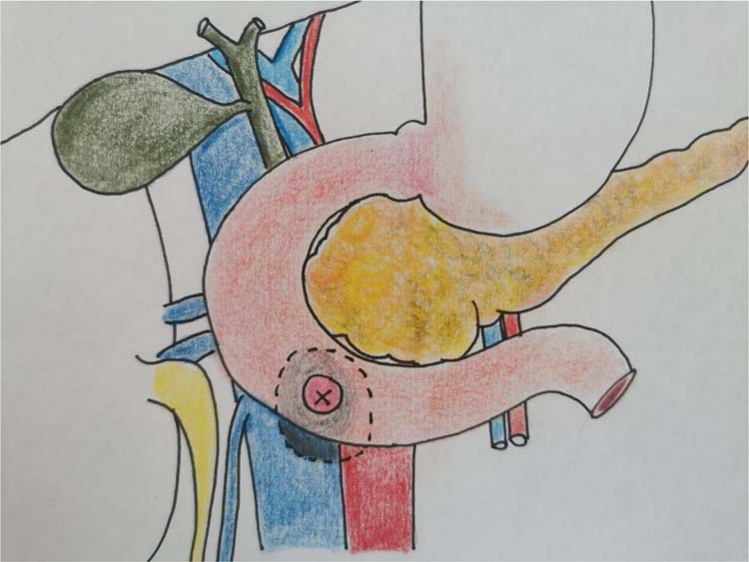
The intraoperative finding confirms the location DCF at the third duodenal portion.

**Figure 4:**
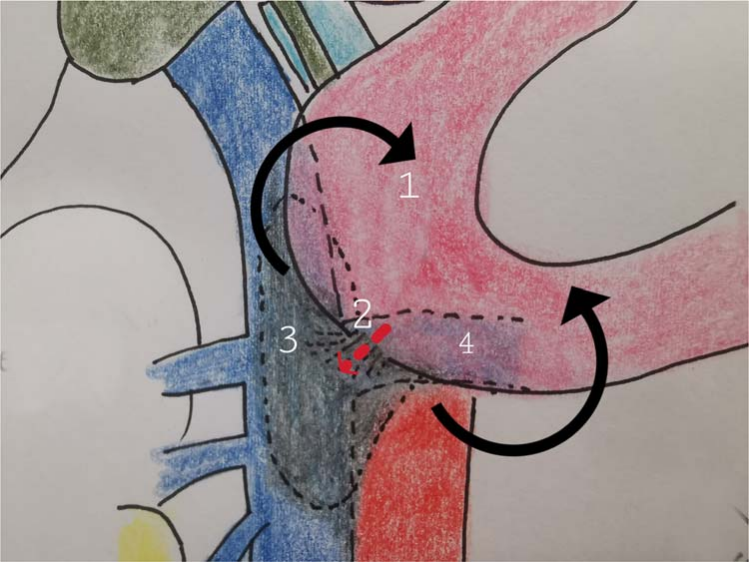
The complete kocherization of the duodenum (1) allowed to better identify the site of the fistula (2) and of the caval thrombus (3) allowing an adequate control of the left renal vein (4).

Therefore, given the persistence of the septic state and the radiological findings, the patient underwent laparotomy. Dense retroperitoneal fibrosis surrounded the IVC and the third section of the duodenum (Fig. 3). After complete mobilization of the duodenum with exposure and preparation of the entire subhepatic inferior caval vein and the two renal veins, the duodenal incision located at the level of the posterior wall of its third portion and the caval vein were sutured (Fig. 4). The cholecystectomy with Kehr drainage placement to protect the bowel suture and a jejunostomy were performed.

The postoperative course, which took place during the first 4 days in the intensive care unit, was uneventful. Enteral feeding through the jejunostomy was undertaken on Day 1st. On the 7th postoperative day, the patient underwent a check of the duodenal suture. The control water-soluble contrast swallow radiography was normal and did not show any leakage; therefore, she began to eat regularly. The patient was discharged on the 14th postoperative day. A first outpatient check-up was performed 7 days after discharge and subsequently 30 days later and no complications were found. At 60 days, Kehr’s drainage was removed, her symptoms had completely disappeared and the patient tolerated a regular diet.

## DISCUSSION

DFC is a very rare pathological condition and this is proved by the scarcity of articles in the literature**.**

Therefore, we performed a complete overview of the literature from 1972 to 2022 and we identified 56 other cases of DFC to assess etiology, clinical presentation, management and outcome summarized in Table 1 [1–26]. The etiologies are trauma, migrating foreign bodies, retroperitoneal surgery followed by irradiation and duodenal ulceration. The latter two are most dangerous, with a mortality of 63.6 and 50%, respectively. Our analysis of the literature shows that diagnosis of DCF poses a major challenge. It requires a high index of suspicion and physician awareness of the risk factors that contribute to its development. The triad of a retroperitoneal or right upper quadrant operation for a malignancy, together with a history of high-dose irradiation and GI hemorrhage, should lead one to suspect DCF.

**Table 1:** Etiology and mortality of cases of DCF [1–26].

Etiology	Patients [*n*, (%)]	Mortality [*n*, (%)]
Migrating Cava Filter [1, 2, 9, 17, 18, 25]	14 (25%)	2 (14.3%)
Abdominal trauma [3]	3 (5.4%)	0
Foreign body [4, 16, 20]	9 (16%)	4 (44.4%)
Peptic Duodenal Ulcer [5, 11, 13, 14, 26]	11 (19.6%)	7 (63.6%)
DCF in association with retroperitoneal tumor and radiotherapy [1, 6–8, 12, 15, 19, 21]	14 (25%)	7 (50%)
DCF in association with retroperitoneal tumor and no radiotherapy [10, 12]	2 (3.4%)	0 (0%)
Bevacizumab treatment [22–24]	3 (5.4%)	3 (100%)
Total	56	23 (41.1%)

The patients were predominantly men (42 men,75% vs 14 women 25%), with a mean age of 50 years (range, 19–73 years). The overall mortality was 41.1% (23 patients).

About half of the DFC were traumatic in origin (26, 46.4%), caused by a penetrating abdominal trauma [3] or the migration of an ingested foreign body (toothpick, chicken or fish bone) [4, 16, 20] or a caval filter, which is one of the most common causes [1, 2, 9, 17, 18, 25]. Such an event is usually a late complication, because the time between caval filter placement and the occurrence of the fistula was on average 6 years (range, 7 days to 11 years) with a mortality mostly found in the ingestion of foreign bodies (four cases) .

Combined right nephrectomy and adjuvant radiotherapy was the second most common etiology (16, 28.6% of the cases) [1, 6–8, 10, 12, 15, 19, 21], suggesting that DCF is mostly related to fibrosis and postradiation damage in the mucosa (a duodenal ulcer is frequently associated with the fistula). We noted two patients who underwent resection of retroperitoneal tumors but did not receive postoperative radiotherapy [10, 12]. Most commonly, DCF was secondary to resection of a right-sided renal or transitional cell carcinoma and six cases of DCF were secondary to retroperitoneal sarcoma resection. Again, it is a long process, because the time between surgery and fistula occurrence was on average 26 months (range, 6–120 months).

Peptic ulcers were responsible for the remaining cases (11, 19.6%) [5, 11, 13, 14, 26]. Only large ulcers accounted DFC, because the mean ulcer diameter was 4.4 cm (range, 1.5–9 cm). However, it is a very serious clinical manifestation with a mortality rate of 44.4%.

Patients with fistulas between the digestive tract and vascular tree classically have the association of sepsis and digestive tract bleeding. Although 30 (69.6%) of the 56 patients with DCF complained of at least one of these signs, only 25 patients (44.6%) demonstrated both septic- and bleeding-related manifestations. Other presentations were nonspecific abdominal pain [9], weight loss [11], fever of unknown origin [2], diarrhea [13], hemorrhagic shock [6], respiratory distress [20] and stroke secondary to cerebral air embolism [7] after gastroduodenoscopy. Because symptoms are so nonspecific, diagnosis is mainly based on the results of radiologic studies, and the abdominal CT is the most sensitive and specific exam. In fact the CT allows you to highlight caval thrombosis, a periduodenal abscess, an incarcerated foreign body or a migrated caval filter. It can also be an indirect means of revealing the DCF by showing hyperdense hepatic and splenic images because of venous passage of ingested contrast [27].

DCFs are life threatening; 23 (41 1%) of the 56 patients died. The mechanism by which the fistula occurs is a prognostic criterion; the mortality rate is high for every etiology, with the exception of migrating caval filters (14.3%). In such cases, a good outcome is probably attributable to early diagnosis; morphologic examinations performed because of abdominal symptoms (mostly pain) were systematically diagnostic by directly showing the migrated device or duodenal perforation [1, 2, 9, 17, 18, 25]. Prognosis is also good in patients with abdominal injury, probably because early surgery is usually performed in such emergency situations [3]. The second prognosis-related criterion is surgery itself; although some deaths occurred despite surgery (because of severe sepsis or bleeding), most of the patients died before they could have an operation. Pancreaticoduodenectomy with gastrojejunostomy and choledochojejunostomy has sometimes been performed [15], but like most authors, we preferred simple suture of the duodenal perforation. Likewise, the IVC is usually sutured and rarely divided or excised [2, 4, 7]. Half the surgical procedures included measures to prevent recurrence of the fistula: epiploic or jejunal patch [2], truncal vagotomy, antrectomy and/or duodenal exclusion [10, 11]. But there are no data on any recurrences. However, morbidity rates remain high, because only 17 (34.7%) of the 49 patients who had surgery eventually became well postoperatively [2–9, 15–21], pointing to the extreme seriousness of the disorder.

This is the complete overview that emerges from an accurate analysis of the literature.

But our case is different as it is associated with the administration of a biological drug such as bevacizumab. Three other similar cases are reported in the literature [22–24]. All of them were women with an average age of 64.7 years (range 58–69 years) with a clinical history of ovarian or cervical cancer who underwent surgery and subsequently radio and chemotherapy with the help of bevacizumab. The presenting clinical picture was similar to our patient, with deterioration of general conditions, sepsis and gastrointestinal bleeding. The clinical manifestations appeared after about 6 months from the beginning of the treatment. But unfortunately for the three cases described [22–24] some type of operation was not performed because of the oncological stage and the serious general clinical conditions and all three died, contrary to our patient who survived the operation. Multiple cases have been described in the literature of intestinal perforation and fistula in patients treated with antiangiogenic drugs [28], with a percentage ranging from 15 to 55.5% of cases but enterocava fistula being a rare find. Related pathophysiological mechanisms are failure of platelet cell endothelial homeostasis, causing submucosal inflammation associated with wall disruption, mesenteric ischemia because of thrombosis and/or vasoconstriction and impaired healing capacity. All this therefore leads to the establishment of a duodenal ulcer and its fistulization with the IVC.

As in our case, a correct multidisciplinary management of these patients was necessary, involving not only the general surgeon, but also the team of vascular surgeons, anesthesiologists for peri- and postoperative management and oncology.
